# Development of Microbial Indicators in Ecological Systems

**DOI:** 10.3390/ijerph192113888

**Published:** 2022-10-26

**Authors:** Fangzhou Ma, Chenbin Wang, Yanjing Zhang, Jing Chen, Rui Xie, Zhanbin Sun

**Affiliations:** 1 Nanjing Institute of Environmental Sciences, Ministry of Ecology and Environment, Nanjing 210042, China; 2 School of Light Industry, Beijing Technology and Business University, Beijing 100048, China

**Keywords:** microbial indicator, monitor, natural ecosystems, artificial ecosystems

## Abstract

Indicators can monitor ecological environment changes and help maintain ecological balance. Bioindicators are divided into animal, plant, and microbial indicators, of which animal and plant indicators have previously been the most researched, but microbial indicators have drawn attention recently owing to their high sensitivity to the environment and their potential for use in monitoring environmental changes. To date, reviews of studies of animals and plants as indicator species have frequently been conducted, but reviews of research on microorganisms as indicator species have been rare. In this review, we summarize and analyze studies using microorganisms as indicator species in a variety of ecosystems, such as forests, deserts, aquatic and plateau ecosystems, and artificial ecosystems, which are contained in wetlands, farmlands, and mining ecosystems. This review provides useful information for the further use of microorganisms as indicators to reflect the changes in different environmental ecosystems.

## 1. Introduction

The ecological environment is the basis for human survival. However, the ecological environment has changed with the intensification of human activities, especially with the deterioration of many environments, which has seriously affected biological habitats [1,2]. Therefore, early detection of ecological environmental changes is important for the sustainable development of the environment. Currently, many indicators are used to monitor environmental change, including physical, chemical, and biological indicators (bioindicators), which can be used to evaluate living or non-living elements in the environment [3,4,5]. Compared with physical and chemical indicators, bioindicator species can comprehensively and dynamically respond to environmental changes and biological effects, which can provide a basis for the accurate assessment of ecological environment changes.

Bioindicators, which commonly refer to species that are widely distributed, are highly sensitive to specific environmental changes and have an indicative function. The abundance and frequency of indicator species are sensitive to environmental changes [6]. Importantly, they must be easy to detect. Biological indicator species are divided into animal, plant, and microbial indicator species according to their taxonomic status. Among the reported animal indicator species, insects are the most dominant, namely, chironomids, ants, and locusts. Chironomids can be used to indicate and monitor water quality in aquatic ecosystems, and ants can indicate the degree of disturbance to the forest [7,8]. Moreover, ants and locusts can be used to indicate the evolution of different types of landscapes or habitats [9,10]. Soil animals such as earthworms or nematodes can be used as important indicators to monitor the restoration of the agricultural, grassland, and forest ecosystems [11,12]. In addition, large animals such as fish, amphibians, and birds can indicate the quality of their related habitats [13,14,15]. Among the reported plant indicator species, phytoplankton is the dominant group of indicator species, which can be used to assess water quality, especially to monitor eutrophication or other water pollution [16]. Bryophytes can be used as indicators to assess the habitat of agro-forestry ecotones and peatlands [17,18]. In addition, large plants can effectively evaluate the forest ecology and soil health. In traditional ecology research, the reported ecological indicator species are mainly animals or plants, as they are macroscopically easy to observe. 

Recent studies have focused on the influence of ecological environment changes on microbial community structure and diversity, which are also necessary for selecting biological indicators [19,20,21]. The advantage of microbial indicators are as follows: (1) Microorganisms are widely distributed in almost all ecological environments, and any microorganism in the environment can be used as an indicator species to assess environmental changes. (2) Microorganisms are highly sensitive to environmental changes. (3) The detection of microbial indicators is relatively easy. The microbial indicators can be detected by the isolation of pure culture or amplicon sequencing in different habitats. In consideration of the potential of microorganisms as indicator species, more studies have been carried out to study the changes in different habitats using microorganisms as indicator species. To date, reviews of studies using animals and plants as indicator species have frequently been conducted, but reviews of studies of microorganisms as indicator species have been rare.

Therefore, the present comprehensive review provides a summary and analysis of research using microorganisms as indicator species to monitor the changes in natural ecosystems such as forests, deserts, aquatic plateaus, and artificial ecosystems (e.g., wetlands, farmland, or mining ecosystems). We also explored the advantages of using microorganisms as indicator species. This review provides useful information for the further use of microorganisms as indicators to reflect the changes in different environments.

## 2. Microbial Indicators in Natural Ecosystems

### 2.1. Forest Ecosystem

The forest ecosystem is one of the main ecosystems of the earth, which plays an important role in maintaining the stability of the biosphere and improving the ecological environment [22,23,24]. Microorganisms, such as bacteria and fungi, are highly sensitive to environmental changes; thus, they are often used as biological indicator species to evaluate interference, natural succession, ecological restoration, and forestry environmental management in the forest ecosystem.

Interference commonly exists in the forest ecosystem, especially under human activity [25,26]. Planting, harvesting, and pollution can influence the microbial community and diversity. For example, different rotation periods of *Eucalyptus* have been shown to impact soil nutrients and enzyme activities as well as the microbial community and diversity. A comprehensive study found that the communities of bacteria phyla such as *Acidobacteria*, *Proteobacteira*, and *Chloroflexi*, as well as fungal phyla such as *Basidiomycota*, *Ascomycota*, and *Zygomycota*, significantly changed in Eucalyptus plantations. This indicates that they can be used as sensitive bioindicators in Eucalyptus plantations to assess soil quality changes [27]. Similar phenomena have been observed in the Atlantic forest ecosystem. The community composition and diversity of arbuscular mycorrhizal fungi (AMF) among six plantation areas with different plants and an Atlantic forest area were investigated. The AMF community composition was different among different plantation areas, indicating that AMF community composition is influenced by land use. Moreover, AMF diversity in the Atlantic forest area was lower than in plantation areas, which indicates that AMF can be used to monitor different plantations [28]. Nitrogen addition could influence AM fungal abundance, richness, and diversity in a forest ecosystem [29]. Additionally, microorganisms can be used to indicate different forest types. In the Indian Himalayan region, oak and pine are the two main evergreen forest types. Dhyani et al. found that the proportion of root fungal endophytes and rhizosphere microorganisms (bacteria, fungi, and actinobacteria) was higher in oak than in pine, indicating that oak can be used as a bioindicator in different forest types in the Indian Himalayan region [30].

Harvesting and anthropogenic pollution are other types of interference in the forest ecosystem [31,32,33]. Hartmann et al. found several potential bioindicators, including ectomycorrhizal fungi, ascomycetes, and actinomycetes, which were more sensitive to forest harvesting compared with other microorganisms and could be used to assess the harvesting and recovery of the forest ecosystem [34]. Moreover, anthropogenic pollution was found to be a threat to mangrove forests. A comparison of the microbial community between anthropogenic pollution and unpolluted mangrove forests found that genus JL-ETNP-Z39 and TA06 exclusively existed in polluted and non-polluted mangrove forests, respectively, and the abundance of *Gemmatimonadetes*, *Cyanobacteria*, *Chloroflexi*, *Firmicutes*, *Acidobacteria*, and *Nitrospirae* was high in the polluted forests. This finding indicates that these microorganisms can be used as bioindicators to monitor the pollution status of the mangrove forest ecosystem. Oil spills are a key pollutant in mangrove ecosystems. A simulated microcosm experiment showed that *Haliea*, *Marinobacterium*, *Marinobacter*, and *Cycloclasticus* were sensitive to oil, indicating that they can be used to monitor oil spills in mangrove forests [35].

In addition to interference, ecological restoration and forestry environmental management are important in the forest ecosystem [36,37]. Soil microorganisms can be used to assess the restoration of forests. Vasconcellos et al. compared soil microorganisms among a native forest and two forests with different restoration periods and found that the abundance of *Solibacteriaceae* and *Verrucomicrobia* considerably differed between the native and restored forests; hence, microorganisms can be used as bioindicators to monitor the soil quality in forest restoration [38]. Agro-forestry environmental management is important for the sustainable development of the forest ecosystem. The microbial community structure can indicate the conversion of the forest ecosystem’s multifunctionality. Soil fungus has exhibited the strongest indicator functions in forest multifunctional conversion in Chinese fir, so it can be proposed as a bioindicator in forest ecosystem management [39]. The microbial community can also influence agricultural management in Amazon forest soils. The abundance of *Acidobacteriota* subgroups 4, 6, and 7 in cropland soil is dramatically higher than that in native forest soil, so these subgroups can be used as bioindicators to assess cropland soil management in the Amazon area [40].

Moreover, soil microorganisms can be used to indicate the succession of forests. Sun et al. selected four typical ecosystems representing the four stages (early, medium, late, and regional climax) of forest ecosystems. The content of total microbes, bacteria, and fungi was higher in stage I than in other stages, and all the microbes had greater abundance in the wet season in stage I [41]. Therefore, these microbes can indicate the succession of forest ecosystems.

### 2.2. Aquatic Ecosystem

Compared with the forest ecosystem, anthropogenic interference is more abundant in the aquatic ecosystem [42,43]. Rivers, lakes, and even oceans can be polluted by metals, eutrophication, or feces. Microbial community composition and diversity have been reported as important bioindicators to monitor the pollution of the aquatic ecosystem.

Microbial community compositions and nutrient pollution in different degrees of disturbed areas (highly and lightly disturbed) in the Songhua River were compared. The results show that the microbial community between the two disturbed areas was considerably different. *Pirellula*, *Synechococcus*, *Alsobacter*, and *Prochlorococcus* could be used to monitor the degradation status of river ecosystems, and *Limnohabitans*, *Flavobacterium*, *Limnobacter*, *Rhodoferax*, *Zavarzinia*, *Pseudarcicella*, and *Pseudorhodobacter* could assess the remediation of river ecosystems [44]. A similar phenomenon was found in the Pearl River Estuary and Milwaukee River Basin. The microbial community-based index was reduced from the upper estuary to the offshore areas of the Pearl River Estuary, which indicates a relationship between eutrophication and fecal pollution. Thus, these microorganisms can be further used to evaluate the impact of human activities on the Pearl River Estuary [45]. Microorganisms can also monitor fecal pollution in the Milwaukee River Basin and be used as bioindicators for assessing water quality [46].

Heavy metals are another important pollutant in the aquatic ecosystem [47,48]. In a metal-polluted aquatic ecosystem, the microbial community and diversity may be highly altered. Bacteria of the genus *Deltaproteobacteria*, *Actinobacteria*, *Coriobacteriia*, *Nitrososphaeria*, *Acidobacteria (Pomacocha)*, *Alphaproteobacteria*, *Chitinophagia*, *Nitrospira*, *Clostridia (Tipicocha)*, and *Betaproteobacteria (Tranca Grande)* were very abundant in lake sediments with Cd and As; thus, these bacteria can be used as bioindicators to monitor heavy metal contamination [49]. Similarly, *Thaumarchaeota*, *Methylophilales*, *Rhodospirillales*, and *Burkholderiales* can be used as bioindicators to indicate acid mine drainage in the aquatic ecosystem [50].

Comprehensive pollution is also common in the aquatic ecosystem. Microbial communities of eight sites representing different impacts of anthropogenic activities from the Yangtze River and the tide of the East China Sea were investigated. The results indicate that methanogens and methanotrophs can be used as bioindicators to assess human activities in the Yangtze Estuary and its coastal area [51]. Similarly, the microbial communities and contaminants of four areas representing pristine, agricultural, industrial, and urban environments were investigated, and it was found that Proteobacteria or Bacteroidetes can be used as useful bioindicators to monitor water quality [52]. River confluences could combine different ecological characteristics, including river pollution. Samson et al. investigated the impact of the confluence of two Indian rivers (Ganges and Yamuna) on microbial communities, and found that fungi of *Aspergillus*, *Penicillium*, *Kluveromyces*, *Lodderomyces*, and *Nakaseomyces* could be used as bioindicators to monitor pollution and eutrophication of river confluences [53].

### 2.3. Desert and Plateau Ecosystem

The desert is one of the harshest natural ecosystems on earth. Yu et al. analyzed the microbial diversity and richness under the succession of the Kubuqi desert ecosystem in three stages: Mobile, semi-mobile, and fixed dunes. The results show that microbial richness was altered among three successional stages and could be used as a bioindicator to assess the successional stages of the desert ecosystem [54]. In the Ningxia Hui Autonomous Region, the microbial community of four desertification stages (i.e., potential desertification, light desertification, severe desertification, and very severe desertification stages) were investigated. The results indicate that *Nitrosomonas*, *Pirellula*, and *Methylobacterium* have the potential to indicate the severe desertification stage [55].

Owing to the special geographical location of plateaus, studies have shown that interference or succession has significance in the plateau ecosystem. Cui et al. investigated the microbial communities in the Loess Plateau in three succession stages, cropland, grassland, and brushland, representing the three stages of agricultural-to-natural ecosystem conversion. The results show that *Firmicutes* was more sensitive than other microbes in the succession stages, which indicates that Firmicutes can be a bioindicator in natural vegetation recovery on the Loess Plateau [56]. In addition to natural succession, interference has been studied on the Loess Plateau. The microbial community and diversity were studied on the Loess Plateau at four interference levels: No grazing, light grazing, moderate grazing, and heavy grazing. The microbial diversity was considerably different among the four interference treatments, and the microbial community composition was most affected by heavy grazing, which indicates that microbial community and diversity may be useful bioindicators to reflect the interference levels on the Loess Plateau [57].

Another experiment was conducted to reflect the ecological characteristics of different distinct landscapes on the Loess Plateau. Liu et al. investigated soil microbial community diversity and richness from eight distinct landscapes on the Loess Plateau, representing forests, grassland, and agricultural lands at the position of 107°39′–109°36′ and the altitude of 415–1633 m. They found that soil fungal diversity and richness were different among samplings, which indicates that the fungal community structure can be used as a bioindicator in the Loess Plateau region [58]. In the Qarhan salt lake area of the Qinghai-Tibet Plateau, Proteobacteria and Halobacteria were dominant in soil, indicating that they can be used as bioindicators to assess the Qarhan salt lake area [59].

## 3. Microbial Indicators in Artificial Ecosystems

### 3.1. Wetland Ecosystem

The wetland ecosystem is generally considered a transition zone between land and aquatic areas in a narrow sense [60]. The wetland ecosystem has been found to be rich in biodiversity but easily destroyed by varieties of pollution. The microbial community can be used as a bioindicator to monitor the pollution in wetland ecosystems. Li et al. studied the relationship between heavy metals and the microbial community in the Huangjinxia nature reserve and found that the abundance of *Nitrospirae*, *Bacteroidetes*, and *Verrucomicrobia* was negatively correlated to the concentration of heavy metals, which indicates that these microorganisms can be used as bioindicators to assess heavy metal pollution in the Huangjinxia nature reserve [61]. Similarly, Roy et al. investigated the relevance of metal and nutrient pollution, and bacterial diversity from wetlands in the urbanizing Pike River, and found that *Fusibacter*, *Aeromonas*, *Arthrobacter*, *Bacillus*, *Bdellovibrio*, and *Chlorobium* could be used to monitor the metal pollution in wetlands in the urbanizing Pike River [62].

Microorganisms could also be used to assess wetland restoration. In a carbonate-rich fen with 10 years of restoration, the microbial community composition and functions were found to be significantly altered, and the microbial component had a potential indicator function for assessing the restoration of carbonate-rich fen [63]. Card et al. investigated the microbial community and structure in different restoration periods in the Prairie Pothole Region (PPR) of Canada and found that specific species in a wetland with a long restoration period were considered bioindicators compared with species in a wetland with a short restoration period [64]. A similar phenomenon has been found in reed rhizosphere microbes. Zheng et al. analyzed the changes in reed rhizosphere microbial diversity and the abundance of restored wetlands and screened those OTUs with a significant difference among different wetland restoration stages as bioindicators to monitor the restoration of wetlands [65].

Similarly, microbial community composition and diversity have been shown to be different among created marshes and native marshes of different ages. The microbial composition of the native marsh has been found to be closest to young marshes. Thus, these distinct microorganisms can be used as bioindicators to assess different types and ages of marshes [66]. Moreover, the microbial community can be effective in monitoring the changes in soil labile organic carbon among the four typical wetland types [67].

### 3.2. Farmland Ecosystem

The farmland ecosystem is the foundation of human survival. The types of operations in farmland, such as effective management, the tillage regime, and fertilization practice, have been found to have an impact on soil microorganisms. The microbial community structure and diversity of three different management practices (conventional, ecological, and intermediate) of cut flower cultures were investigated, and it was found that the bacterial community structure differed considerably among the three management practices, indicating that it can effectively monitor the soil quality change by different management practices of cut flower cultures [68]. In addition to management practices, the tillage regime has also been found to influence the soil microbial community. Sale et al. studied the influence of different tillage regimes in Sissle Valley on the soil AMF community structure and richness, and the results indicate that AMF species exclusively existing in each tillage regime can be used as bioindicators [69]. Different fertilization practices have also been found to impact the community composition and diversity of soil microorganisms and plant endophytes. Long-term (34 years) fertilization practices were conducted using chemical fertilizer and organic manure in the North China Plain. The community composition and diversity of soil microorganisms and wheat endophytes were investigated under the two fertilization practices. The results show that the application of chemical fertilizer and organic manure had an impact on the community composition and diversity of soil microorganisms and wheat endophytes [70,71]. In the soil microbial community, the abundance of *Fusarium* and *Penicillium* was different between the two types of fertilizers, and in the wheat endophytic community, the abundance of *Brevundimonas* was higher in organic manure than in chemical treatment, which indicates that these microorganisms can be used as bioindicators in different fertilization practices.

Interference has also been found in farmland. Fires have been found to alter the soil characteristics and microbial community. A laboratory experiment was conducted to mimic the burning of crop residues. The results show that the abundance of Firmicutes was highly increased and could be used to monitor environmental changes such as the fire impact [72]. Farmland restoration is also important for the farmland ecosystem. Zhang et al. investigated the impact of 14-year field trials of degraded cropland among restoration treatments on the microbial community. The results show that compared with the treatment of bare land soil (without biomass), the abundance of *Bacillus* (Firmicutes) and *Cyanobacteria* was dramatically reduced in natural grassland and alfalfa cropland (with biomass) treatments, indicating that both bacteria can be used as bioindicators to assess farmland degradation [73]. In a grassland ecosystem, compared with soil bacteria, fungi are more sensitive to grassland degradation, which suggest potential bioindicators for grassland ecosystem management [74]. 

### 3.3. Mining Ecosystem

The mining ecosystem is mainly associated with human exploitation. Microorganisms can be used as bioindicators to evaluate pollution and ecological restoration in the mining ecosystem. Pollution is the most influential threat to the environment in the mining ecosystem. Thus, effective monitoring is important for the mining ecosystem. Xiang et al. investigated the soil microbial community of Guangxi manganese mining at different distances. The results show that the abundance of Firmicutes and Bacteroides was dominant in the center of the manganese mining area, indicating that they can be used to monitor the pollution of the manganese mining area [75]. In addition, the impact on the microbial community composition and diversity in the Jiaopingdu Cu mine wastewater treatment plant was also investigated. Compared with fungus, the bacteria abundance and taxa were more sensitive to environmental alteration, indicating that they can be used as bioindicators to monitor Cu mine pollution [76]. Similarly, metal pollution in alkaline copper mine drainage altered the bacterial community composition, diversity, and richness. Thus, *Thiovirga* and *Symbiobacterium* can be used as bioindicators to assess pollution [77].

Microorganisms could also be used to assess mining restoration [78]. Ezeokoli et al. investigated the diversity and community structure of AMF in rhizosphere soil and plant roots during different post-coal mining reclamation periods. The community structure of AMF had a dramatic difference in unmined and reclamation soils, as well as between younger and older reclaimed areas. *Acaulospora*, *Diversispora*, *Paraglomus*, and *Scutellospora*, with different abundance levels, were suggested to monitor ecosystem restoration [79]. Chen et al. analyzed the microbial abundance, community diversity, and multifunctionality in a coal mine dump and found that *Nitrosomonadaceae*, *Rhizobiales*, and *Acidimicrobiales* could be used to assess the ecological restoration of coal mine dumps [80]. The fungal community diversity in a rehabilitated mine site of different periods was analyzed. Fungal richness was significantly higher in rehabilitated mine sites than in nonmined sites [81]. In addition, the fungal community composition was impacted by different rehabilitation times, which indicates that soil fungal diversity can be used as an effective bioindicator to assess soil restoration in mined sites. The microbial community was investigated between artificial restoration areas and naturally regenerated areas of the Shendong coal mine, and it was found that the microbial composition and diversity were considerably different in the two areas, indicating that the microbial composition can be used to monitor artificial restoration in mining areas [82].

## 4. Conclusions

Indicator species are important to monitor environmental change in ecological systems. To date, animals and plants have been the most common indicator species, mainly because they are easy to observe and measure. Recently, microorganisms, mainly bacteria and fungi, have been studied as bioindicators in different ecosystems due to their high sensitivity to environmental changes. This review has focused on comprehensive research on using microorganisms as bioindicators in a variety of ecosystems, including natural and artificial ecosystems. This review has provided useful information for the further use of microorganisms as indicators to reflect changes in different ecosystems. Further studies on the use of microorganisms as bioindicators should extend the number of microbial species assessed for their bioindicator function. In addition, microbial indicators could be used in more types of ecosystems.

## Data Availability

Not applicable.
